# Extracting Physician Group Intelligence from Electronic Health Records to Support Evidence Based Medicine

**DOI:** 10.1371/journal.pone.0064933

**Published:** 2013-05-29

**Authors:** Griffin M. Weber, Isaac S. Kohane

**Affiliations:** 1 Information Technology, Harvard Medical School, Boston, Massachusetts, United States of America; 2 Interdisciplinary Medicine and Biotechnology, Beth Israel Deaconess Medical Center, Boston, Massachusetts, United States of America; 3 Center for Biomedical Informatics, Harvard Medical School, Boston, Massachusetts, United States of America; 4 Medicine, Partners HealthCare System, Boston, Massachusetts, United States of America; 5 Pediatrics, Children's Hospital Boston, Boston, Massachusetts, United States of America; University of Vermont, United States of America

## Abstract

Evidence-based medicine employs expert opinion and clinical data to inform clinical decision making. The objective of this study is to determine whether it is possible to complement these sources of evidence with information about physician “group intelligence” that exists in electronic health records. Specifically, we measured laboratory test “repeat intervals”, defined as the amount of time it takes for a physician to repeat a test that was previously ordered for the same patient. Our assumption is that while the result of a test is a direct measure of one marker of a patient's health, the physician's decision to order the test is based on multiple factors including past experience, available treatment options, and information about the patient that might not be coded in the electronic health record. By examining repeat intervals in aggregate over large numbers of patients, we show that it is possible to 1) determine what laboratory test results physicians consider “normal”, 2) identify subpopulations of patients that deviate from the norm, and 3) identify situations where laboratory tests are over-ordered. We used laboratory tests as just one example of how physician group intelligence can be used to support evidence based medicine in a way that is automated and continually updated.

## Introduction

In evidence-based medicine (EBM), clinical practice guidelines are driven by expert consensus, which is typically based on review of the literature, clinical experience, and outcomes analyses [1], [2]. A major challenge of EBM is the effort and cost needed to keep the knowledge of clinical practice up to date across an ever-widening array of diagnostic and therapeutic options [3]. One way to approach this problem is through analysis of the large amounts of data collected in electronic health records (EHR) [4]. Usually the variable being examined in these datasets is a patient outcome, such as survival [5]. However, in this study we will demonstrate that EHRs not only contain information about patient outcomes, but they also provide insight to providers' knowledge of their patients' state of health, which can also be used in generating EBM guidelines. We will do this in the context of laboratory tests. Instead of looking at the results of the tests, we will examine when physicians ordered the tests. Whereas the result of a test is a direct measure of one maker of a patient's health, a physician's decision to order a test is based on multiple factors including past experience, available treatment options, and information about the patient that might not be coded in the EHR.

Specifically, we will measure laboratory test “repeat interval”, defined as the amount of time it takes for a physician to repeat a test that was previously ordered for the same patient. For example, if a white blood cell count (WBC) test is ordered for a patient, and the next time that patient has a WBC test is seven days later, then the repeat interval is seven days. The physician ordering the repeat test is not necessarily the same person who ordered the previous test, but could presumably access the result of the previous test through the EHR. By examining these repeat intervals in aggregate over large numbers of patients, we can quantify physician behavior and observe how it varies under different conditions. To demonstrate how this can be used for EBM, we will use the laboratory test repeat intervals from the EHRs of two large and independent hospitals in the Boston area to answer three questions: Firstly, can collective physician laboratory test-ordering behavior, which we call physician “group intelligence”, be used to define what it means for a laboratory test result to be “normal”? Secondly, can subpopulations of patients be identified when their physicians' behavior differs from the norm? Finally, can physician group intelligence be used to identify situations where laboratory tests are over-ordered?

## Methods

### Data Sources

The data used for this study were laboratory test results contained within the Partners Research Patient Data Repository (RPDR), a large clinical database, which combines data from Brigham and Women's Hospital (BWH) and Massachusetts General Hospital (MGH) [6]–[8]. From an initial dataset, which included 3,534,666 patients with 465,313,629 laboratory test results between 1/1/1986 and 6/30/2004, we extracted two datasets: (1) Firstly, we obtained a random sample of 100,000 repeat intervals for each of the 97 different laboratory tests listed in Table 1 (9.7 million repeat intervals). Other laboratory tests were excluded either because they have fewer than 100,000 occurrences, or there are known problems with how the data are recorded. Although there are 4,926 tests in the RPDR, these 97 represent 71% of all test results because they are the ones most frequently ordered. (2) Secondly, we obtained a random sample of 1,000,000 repeat intervals for white blood cells (WBC), which indicated the patient age in days at the time of the tests and whether the tests were performed in inpatient or outpatient settings. The laboratory test dates in the RPDR are typically the dates when the results are ready, rather than when the specimens were obtained or when the results were read. The datasets may be requested by registration and submission of a Data Use Agreement at http://www.i2b2.org/Publication_Data/.

**Table 1:** Summary of repeat intervals for 97 laboratory tests.

Laboratory Test (LOINC Name)	LOINC Code	Median Repeat Interval	Min Bin Median Repeat Interval	Max Bin Median Repeat Interval	Ratio Bin Median Repeat Interval	StDev Repeat Interval	Entropy	Category
pH SerPl-sCnc	2753-2	0.121	0.059	0.150	2.541	111.8	0.722	BGB
Base Excess BldA-sCnc	1925-7	0.122	0.078	0.176	2.248	144.1	0.881	BG
O2 Satn from pO2 Fr Bld	2713-6	0.123	0.065	0.176	2.691	135.6	0.881	BGB
pO2 Bld Qn	11556-8	0.124	0.027	0.168	6.205	117.4	0.811	BGB
pCO2 Bld Qn	11557-6	0.124	0.063	0.150	2.400	127.5	0.610	BGB
Inhaled O2 rate	3151-8	0.136	0.059	0.235	3.976	43.0	1.279	GB
Ca-I Bld-sCnc	1994-3	0.157	0.082	0.169	2.068	156.8	0.286	BGB
HCO3 Bld-sCnc	1959-6	0.208	0.118	0.253	2.147	76.8	0.881	BG
Ca-I SerPl-sCnc	1995-0	0.218	0.167	0.258	1.550	169.2	0.722	BG
Gentamicin SerPl-mCnc	35668-3	0.379	0.076	2.418	31.945	199.4	1.915	BG
CK MB SerPl EIA-cCnc	6773-6	0.402	0.342	0.472	1.379	368.9	0.971	BGB
CK/CK MB SerPl-cRto	2158-4	0.424	0.383	0.463	1.208	337.7	0.934	GBGB
Osmolality SerPl Qn	2692-2	0.444	0.278	0.850	3.060	269.4	1.458	GB
Troponin T SerPl-mCnc	6598-7	0.485	0.440	0.539	1.224	116.6	0.000	BG
CK SerPl-cCnc	2157-6	0.642	0.424	1.017	2.402	400.8	0.881	GB
Troponin I SerPl-mCnc	10839-9	0.741	0.439	19.857	45.244	284.7	1.882	GB
aPTT Plas Qn	5898-2	0.927	0.378	1.270	3.356	346.7	1.141	GB
Potassium SerPl-sCnc	2823-3	0.958	0.444	0.987	2.224	291.7	0.286	BGB
Vancomycin SerPl-mCnc	20578-1	0.974	0.144	1.519	10.519	174.3	0.848	BG
Magnesium SerPl-sCnc	11554-3	0.977	0.483	1.042	2.157	284.7	0.286	GB
CO2 SerPl-sCnc	2028-9	0.988	0.408	1.144	2.801	311.5	0.748	BGB
Glucose SerPl-mCnc	6777-7	0.996	0.292	24.978	85.437	332.3	2.528	GB
Sodium SerPl-sCnc	2951-2	0.997	0.426	1.846	4.329	310.6	0.748	BGB
Anion Gap3 SerPl-sCnc	10466-1	1.000	0.829	1.015	1.224	307.1	0.000	BGB
Hgb Bld-mCnc	718-7	1.010	0.391	33.962	86.865	324.8	2.461	BG
Hct Fr Bld Auto	4544-3	1.026	0.490	40.188	81.970	336.9	2.595	BG
PT PPP Qn	5902-2	1.035	0.974	3.119	3.202	332.6	1.054	GBG
Chloride SerPl-sCnc	2075-0	1.049	0.600	1.876	3.127	330.5	0.748	BGB
INR PPP Qn	6301-6	1.053	0.982	6.998	7.127	235.8	1.154	GBG
BUN SerPl-mCnc	3094-0	1.068	0.722	2.994	4.145	338.5	1.439	BGB
Creat SerPl-mCnc	2160-0	1.090	0.892	2.193	2.460	333.4	0.993	BGB
RDW RBC Auto-Rto	788-0	1.103	0.987	42.922	43.495	337.1	1.871	GB
MCH RBC Qn Auto	785-6	1.204	1.052	2.788	2.650	351.9	0.992	GBG
MCV RBC Qn Auto	787-2	1.205	1.052	2.754	2.618	347.8	1.076	GBG
WBC # Bld Auto	6690-2	1.213	0.769	15.403	20.037	351.6	1.923	BGB
Phenytoin SerPl-mCnc	3968-5	1.217	1.122	1.944	1.733	201.3	0.610	GBGB
RBC # Bld Auto	789-8	1.217	0.796	43.963	55.242	347.5	2.361	BG
Platelet # Bld Auto	777-3	1.225	0.735	7.033	9.573	349.8	2.009	BGB
MCHC RBC Auto-mCnc	786-4	1.231	1.040	2.024	1.947	355.6	0.993	BGB
Calcium SerPl-sCnc	2000-8	1.644	0.736	35.068	47.640	390.6	2.458	BG
Digoxin SerPl-mCnc	10535-3	1.960	1.001	3.143	3.139	263.6	1.157	GB
Phosphate SerPl-mCnc	2777-1	1.965	1.003	5.594	5.574	384.3	1.971	BGB
Lipase SerPl-cCnc	3040-3	1.974	0.999	5.031	5.038	427.1	1.839	BGB
Metamyelocytes Fr Bld Manual	740-1	2.426	1.053	4.111	3.902	289.6	1.720	GB
Amylase SerPl-cCnc	1798-8	2.446	0.989	10.447	10.564	529.3	2.204	BGB
Myelocytes Fr Bld Manual	749-2	2.456	1.043	4.278	4.101	277.0	1.720	GB
Neuts Seg Fr Bld Manual	769-0	2.673	1.067	5.905	5.536	365.7	2.195	BGB
Monocytes Fr Bld Manual	744-3	2.760	1.092	4.041	3.699	358.7	1.788	BGB
Basophils Fr Bld Manual	707-0	2.776	2.021	6.968	3.448	359.8	1.076	BG
Lymphocytes Fr Bld Manual	737-7	2.790	1.060	11.269	10.627	390.8	2.409	BGB
Eosinophil Fr Bld Manual	714-6	2.846	1.886	5.990	3.176	367.6	1.713	BG
Neuts Band # Bld Manual	763-3	2.924	1.028	8.065	7.842	172.1	2.285	GB
Estradiol SerPl-mCnc	2243-4	2.988	1.051	13.929	13.248	232.2	1.981	GBG
Atypical Lymphs Fr Bld Manual	735-1	3.040	2.123	5.901	2.780	416.0	1.739	BG
Neuts Band Fr Bld Manual	764-1	3.894	1.020	42.122	41.291	396.5	2.771	GBGB
Cyclosporin Bld-mCnc	3520-4	5.970	2.974	11.236	3.778	92.6	1.533	GB
LDH SerPl-cCnc	2532-0	8.132	1.031	33.894	32.889	368.5	2.984	BGB
HCG SerPl-sCnc	2119-6	8.895	2.992	276.063	92.256	531.9	2.704	GBG
Globulin Ser-mCnc	2336-6	10.912	1.037	28.033	27.038	458.4	2.409	BGB
Prot SerPl-mCnc	2885-2	15.105	0.982	102.826	104.717	448.8	2.566	BG
Retics/100 RBC Fr	4679-7	17.717	6.372	41.006	6.436	516.0	2.522	BGB
Bilirub SerPl-mCnc	1975-2	19.547	0.956	47.202	49.362	442.4	2.458	GB
Urate SerPl-mCnc	3084-1	20.169	1.050	44.928	42.789	415.0	2.823	BGB
Albumin SerPl-mCnc	1751-7	20.757	1.118	128.127	114.598	444.0	3.141	BG
ALP SerPl-cCnc	6768-6	27.978	1.289	70.348	54.580	443.2	2.561	GB
Basophils Fr Bld Auto	706-2	28.043	2.110	52.266	24.774	410.5	2.804	BGB
Eosinophil Fr Bld Auto	713-8	28.081	2.555	52.915	20.711	410.3	2.522	BGB
ALT SerPl-cCnc	1742-6	28.414	1.110	58.151	52.369	430.1	2.361	BGB
Eosinophil # Bld Auto	711-2	28.920	2.501	60.021	24.002	409.5	2.746	BGB
Basophils # Bld Auto	704-7	29.528	4.877	52.957	10.858	411.1	2.571	BGB
AST SerPl-cCnc	1920-8	30.291	1.055	78.035	73.977	431.8	2.461	BGB
Monocytes Fr Bld Auto	5905-5	34.410	5.740	56.119	9.776	464.5	2.384	BGB
Neutrophils Fr Bld Auto	770-8	34.686	1.885	92.702	49.168	468.3	3.141	GB
Lymphocytes Fr Bld Auto	736-9	34.826	1.797	114.231	63.560	464.9	2.946	BG
Neutrophils # Bld Auto	752-6	34.932	1.768	75.975	42.971	457.9	2.461	BGB
Monocytes # Bld Auto	742-7	35.744	3.636	83.958	23.090	470.4	2.358	BGB
Lymphocytes # Bld Auto	731-0	37.134	1.963	99.138	50.498	462.7	2.622	BG
PMV Bld Qn	28542-9	37.998	10.019	67.001	6.687	254.6	2.119	BGB
Sp Gr Ur Qn Strip	5811-5	49.284	33.625	81.578	2.426	581.0	1.782	GBGB
RBC # UrnS HPF	5808-1	49.988	12.815	83.504	6.516	619.8	2.115	GB
pH Ur Strip-sCnc	5803-2	51.020	36.494	70.001	1.918	594.1	1.295	BGB
Trigl SerPl-mCnc	2571-8	108.380	42.058	225.953	5.372	498.3	2.121	GB
ESR Bld Qn Westrgrn	4537-7	125.983	21.147	246.166	11.641	672.5	2.085	GB
Cholest SerPl-mCnc	2093-3	127.327	1.059	204.759	193.346	503.5	1.933	BGB
Hgb A1c Fr Bld	4548-4	132.945	113.022	216.175	1.913	328.8	1.076	GB
Mean Glucose Bld gHb Est-mCnc	27353-2	137.837	120.280	266.906	2.219	325.6	1.076	GB
Ferritin SerPl-mCnc	2276-4	151.261	34.569	213.974	6.190	622.9	1.457	GB
T4 SerPl-mCnc	3026-2	154.974	47.040	202.118	4.297	629.6	1.357	BGB
Iron SerPl-mCnc	2498-4	160.883	83.993	266.211	3.169	628.1	1.846	BGB
TIBC SerPl-mCnc	2500-7	166.066	27.024	248.118	9.182	649.0	1.717	BG
TSH SerPl-aCnc	3016-3	232.133	55.991	368.103	6.574	578.2	1.579	BGB
Cholest/HDLc SerPl-mRto	9830-1	247.117	131.251	367.034	2.796	348.0	1.234	GB
VLDLc SerPl-mCnc	2092-5	253.763	175.665	363.871	2.071	563.9	0.993	GB
B-LP SerPl Calc-sCnc	14815-5	264.197	166.951	346.086	2.073	578.8	0.881	BGB
LDLc SerPl-mCnc	2090-9	273.672	172.881	343.069	1.984	517.4	0.881	BGB
HDLc SerPl-mCnc	2086-7	276.331	146.957	370.070	2.518	528.6	1.188	BG
PSA SerPl-mCnc	2857-1	350.024	75.940	380.037	5.004	392.9	1.076	BGB

Listed for each test are the LOINC (Logical Observation Identifiers Names and Code) name and code; the median repeat interval and standard deviation for all 100,000 repeat intervals; the minimum and maximum median repeat intervals of the 20 value bins and their ratio; the entropy; and the category: “bad-good” (BG), “bad-good-bad” (BGB), “good-bad” (GB), “good-bad-good” (GBG), and “good-bad-good-bad” (GBGB). Repeat intervals are given in days.

### Defining normality

Reference ranges of laboratory test values are defined by sampling a healthy population and recording the upper and lower n^th^ percentiles [9]–[11]. There are numerous challenges with determining these ranges and in using them for clinical decision-making. Many factors such as age, sex, and sampling bias can influence these values; it can be difficult to identify healthy individuals; and there is disagreement over which statistical techniques and percentiles to use [12]–[15]. Furthermore, it is unclear how useful reference ranges are in clinical decision-making since there is a distinction between a reference limit and the value that will actually change a physician's clinical decision [16]–[19]. The latter is based not on healthy population percentiles, but rather the types of clinical actions that are available to the physician and his or her clinical knowledge, prior experience, and intuition. Can we quantify this to define a new robust measure of laboratory test value normality that reflects clinical expertise?

We defined repeat interval as the amount of time it took for physicians to repeat the same test in the same patient. A repeat interval consists of two tests—an initial test and a repeat test. In this study, we looked at the relationship between the *result* of the initial test and *when* the repeat test is ordered. To study this relationship, for each of the 97 laboratory tests we partitioned the 100,000 repeat intervals into 20 equal-size bins based on the result of the initial test. For example, the first bin contains the 5,000 repeat intervals with the smallest initial test result values, and the 20^th^ bin contains the 5,000 repeat intervals with the highest initial values. For each bin, we calculated the median repeat interval duration and the 25^th^ and 75^th^ percentiles. We did not use the result of the repeat test in this study—we only measured the amount of time that had elapsed since the initial test. Note how this differs from traditional EBM studies, in which physicians perform interventions, and then the patient outcomes are measured. In this study, we start with data about the patients (their initial laboratory test results), and then measure the interventions chosen by their physicians (the time until the test was repeated). In other words, we are examining the physicians as a way of indirectly learning more about the patients.

In the first part of this study, we used repeat intervals to examine normality in laboratory tests. Whereas laboratory test reference ranges suggest there are only two states of patient health, normal and abnormal, we hypothesized that repeat intervals would reveal more subtle patterns that demonstrate the variability among patients and the different clinical contexts in which they are seen.

### Identifying subpopulations

To determine if we can automatically identify the various factors that can influence physician behavior, such as patient demographics and clinical settings, we calculated the median repeat intervals for white blood cells (WBC) for different pediatric age groups and for inpatient vs outpatient visits. If these subpopulations indeed represent distinct patient states that have different clinical meaning, then differences in normative behavior might be detectable.

### Measuring informativeness

The initial test result may or may not influence when the repeat test is ordered. We used entropy as a measure of how much the median repeat interval varies across the 20 bins for each test. If all 20 median repeat intervals are equal, then the initial test result provides no information towards predicting when the repeat test will be ordered, and the entropy is therefore zero. Because physician behavior is not being affected by the result of the test, we hypothesize that some tests with low entropy are being over-ordered. In contrast, tests whose initial result has a greater influence over physician behavior will have higher entropy, suggesting that those tests are more informative.

**Figure 1:**
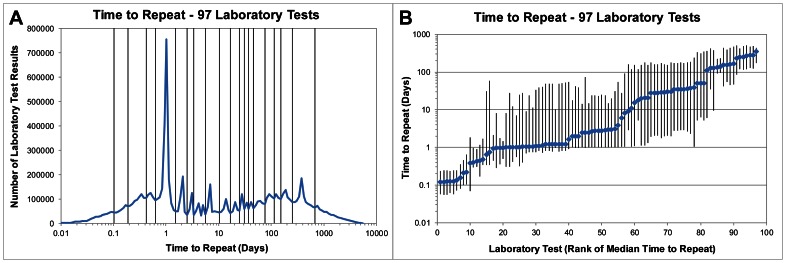
Repeat intervals for 97 common laboratory tests. (a) Frequency distribution of repeat intervals for all labs. Vertical bars indicate the boundaries used in the entropy calculations to convert repeat intervals to one of 20 discrete categories. (b) Median repeat interval for each of 97 tests. Vertical bars indicate the 25th and 75th percentiles.

In order to calculate entropy, we first discretized the median repeat interval for each laboratory test's 20 value bins by mapping it to one of 20 frequently observed time periods (Table 2). These time periods were determined by combining the repeat intervals for all 97 laboratory tests and noting from its frequency distribution that there are approximately 20 peaks (Figure 1a). The points between the peaks with the fewest repeat intervals were chosen as the boundaries of the time periods. This ensured that most repeat intervals would be near the center of a time period rather than at the boundary, thus making the results less sensitive to the precise location of the time period boundaries. Entropies were then calculated using the equation -Sum[p(x)*log_2_(p(x))] where p(x) is the fraction of a laboratory test's 20 value bins whose median repeat intervals fall within time period x. For example, if a laboratory test has 10 value bins whose median repeat intervals fall within time period 6 (2 days), 5 value bins that fall within time period 4 (12 hours), and 5 value bins that fall within time period 7 (3 days), then the entropy is −[0.5*log_2_(0.5)+0.25*log_2_(0.25)+0.25*log_2_(0.25)] = 1.5.

**Table 2:** Entropy time periods.

Time Period	Description	Start (Days)	Peak (Days)
1	2 hours	0.000	0.093
2	4 hours	0.103	0.170
3	8 hours	0.188	0.342
4	12 hours	0.418	0.510
5	24 hours	0.624	1.028
6	2 days	1.533	2.070
7	3 days	2.528	3.088
8	4 days	3.413	4.169
9	7 days	5.627	6.873
10	2 weeks	10.253	13.840
11	3 weeks	16.905	20.648
12	4 weeks	25.219	27.872
13	5 weeks	30.803	34.042
14	6 weeks	37.622	41.579
15	2 months	45.952	62.029
16	3 months	75.763	92.536
17	4 months	113.024	124.911
18	6 months	152.567	186.345
19	1 year	251.540	375.253
20	2 years	683.756	755.667

Listed are the start of each time period and the most common repeat interval (peak).

## Results

Table 2 and Figure 1a show that the frequency of 9.7 million repeat intervals across the 97 tests has approximately 20 peaks, with 24 hours being the most common, followed by 2 days, 1 year, 7 days, and 6 months. When looking at individual laboratory tests, Table 1 and Figure 1b show that the median repeat interval can range from as small as 3 hours for blood gases to as large as year for cholesterol and prostate-specific antigen (PSA), with a large variance for most tests. However, the repeat intervals can be highly dependent on the initial value of the test as well as the patient population and clinical setting. The next three sections describe this relationship by testing three hypotheses.

### Can physician group intelligence derive knowledge that all physicians already know, but can be difficult to quantify?

**Figure 2:**
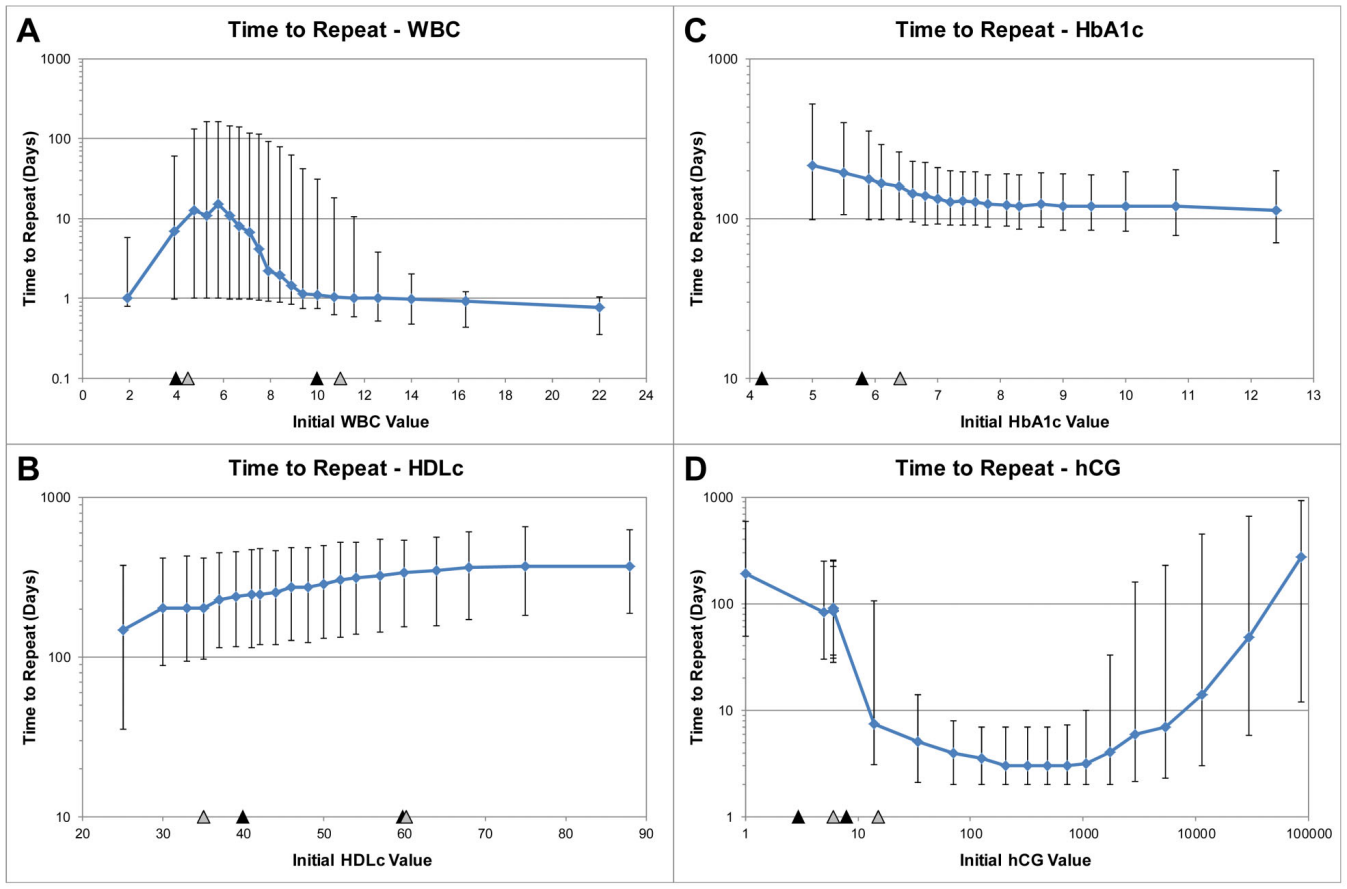
The median repeat interval for different initial (a) WBC, (b) HDLc, (c) HbA1c, and (d) hCG values. Error bars represent the 25th and 75th percentiles. Triangles indicate reference values for BWH (black) and MGH (gray).

The reference ranges for white blood cell count (WBC) in adult patients at BWH and MGH are 4.0–10.0 and 4.5–11.0, respectively [6], [7]. In Figure 2a, which illustrates the repeat intervals for WBC, we can see a complex relationship between the initial WBC value and when physicians order a second WBC test. In general, the repeat interval for WBC is larger within the hospital reference ranges (indicated by markers on the horizontal axis) than outside. However, it is not a binary response. Rather, there is a continuum, with a maximum median repeat interval of almost two weeks at an initial WBC value of 6, and gradually decreasing at larger or smaller values. As seen in Figure 2b and Figure 2c, a similar pattern exists for other tests, such as high-density lipoprotein (HDLc) and hemoglobin A1c (HbA1c), where the largest repeat intervals occur when the initial test results are within the hospital reference ranges, and the intervals decrease the further outside those ranges.

The vertical bars in Figure 2 represent the 25th and 75th percentiles of repeat intervals. The initial test result not only affects the median repeat interval, but it also greatly affects the variance. If we think about an initial test result being followed by a large median repeat interval as a “good” test result, and an initial test result being followed by a small median repeat interval as a “bad” test result, then the amount of variance corresponds to the degree of consensus among physicians on whether a particular test result is “good” or “bad”. For example, on average, a WBC of 6 is “good”, but the large variance means that other information is needed to determine the patient's state of health. At the upper value of the reference range (10.0–11.0), the repeat interval is smaller, but there is still large variability. However, once the WBC is greater than 16, then there is agreement among physicians that the result is “bad”.

Laboratory tests can be classified according to how their repeat intervals vary with different initial values. Although WBC is “good” in mid-range values and “bad” at the low and high extremes (“bad-good-bad”, or “BGB”), the repeat intervals for HDLc are largest at high values (“BG”), and the repeat intervals for HbA1c are largest at low values (“GB”). Table 1 shows that most laboratory tests fall into one of these three categories, with 44 BGB tests (e.g., sodium and glucose), 19 BG tests (e.g., hematocrit and vancomycin), and 24 GB tests (e.g., bilirubin and erythrocyte sedimentation rate (ESR)). An exception is human chorionic gonadotropin (hCG), which has not one, but two “good” states (“GBG”) depending on whether the patient is pregnant (Figure 2d).

Although we are not arguing that this method should replace the standard way of determining laboratory test reference ranges, we want to highlight how remarkable it is that repeat intervals alone, without any additional information about the patients' health, can be used to derive physician consensus around what it means for a test result to be “normal”. In other words, we can use physician group intelligence to quantify the significance of different test results and determine the values that require immediate action.

### Can group intelligence capture the knowledge of subsets of physicians that treat specific patient populations?

**Figure 3:**
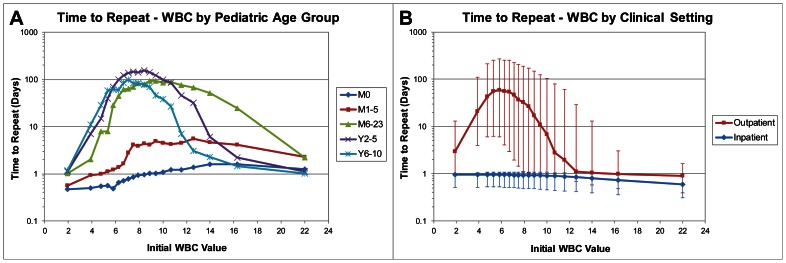
Factors that affect repeat interval, including (a) age and (b) inpatient vs outpatient setting. Error bars represent the 25th and 75th percentiles.

Normality as defined by physician behavior can vary greatly with different subpopulations. In neonates, for example, the typical WBC is higher than in adult populations. Figure 3a shows that physicians adjust their ordering behavior for this, with a peak time to repeat for patients less than 1 month old at a WBC of 16.3 (58,121 repeat intervals). As pediatric patients age, the “ideal” WBC value decreases and the maximum repeat interval increases. For patients 1–5 months the preferred value is 12.6 (16,237 repeat intervals), and for patients 6–23 months the preferred value is 8.9 (32,556 repeat intervals). The median time to repeat of WBC is at a maximum of 153 days when patients are between 2–5 years old (33,666 repeat intervals). Beyond this age, physician behavior mimics that seen throughout adulthood (38,051 repeat intervals). However, while the preferred WBC remains consistent until old age, the repeat intervals decrease for all values in elderly populations.

### Can group intelligence identify inconsistencies in clinical behavior and situations where the frequency of ordering laboratory tests can be reduced?

Figure 3b shows that physician ordering behavior for WBC also changes when patients are in an inpatient setting compared to when they are relatively healthy in an outpatient setting. In both cases, the maximum repeat interval is at a WBC value of about 6. However, that interval is 22.9 hours for inpatients (365,769 repeat intervals) and 59.1 days for outpatients (481,591 repeat intervals). Thus, the same laboratory test result can have a dramatically different effect on clinical decisions depending on physician's perceived state of the patient. It might also suggest that hospital guidelines in an inpatient setting influence ordering behavior in ways that are counterintuitive to physicians' true estimate of risk.

The extent to which the initial value of a laboratory test affects the repeat interval can indicate how informative that test is. For nearly all 97 laboratory tests studied, the initial value does indeed influence the repeat interval greatly (Table 1). For example, the ratio between WBC's best bin's repeat interval (15.4 days) and the worst (0.77 days) is 20-fold. There was at least a 2-fold difference in 87 tests, a 10-fold difference in 35 tests, a 50-fold difference in 13 tests, and a more than 100-fold difference in three tests (serum protein, albumin, and cholesterol). However, this does not tell the full story. A test whose repeat interval is the same in nearly all cases except for the most extreme values might provide less information to a physician, on average, than a test whose repeat intervals vary across the full range of values for that test. This can be quantified using entropy.

Of the 97 tests, albumin and neutrophil fraction had the highest observed entropies (3.141), meaning that their values, more than any other tests, had the greatest influence on physician behavior (Table 1). There are several explanations for why the entropy can be low for certain laboratory tests: a) they can be routinely ordered as part of a hospital protocol (e.g. Troponin T has zero entropy), b) they are ordered automatically as part of a panel but are not generally the reason for which the panel was ordered (e.g. mean corpuscular volume (MCV) in a complete blood count (CBC) has an entropy of 1.076), or c) they are part of a screening protocol in which the vast majority of the test results are normal (e.g. prostate-specific antigen (PSA) has an entropy of 1.076 because 75% of its values are less than 3.6 and are not repeated for a one year).

## Discussion

We introduced this study by enumerating three questions that we sought to answer, at least preliminarily in a study of two large academic hospitals. We have shown that we can use collective physician behavior to identify normal ranges that correspond to the published normal ranges used in these institutions but without the threshold effect of strict limits and instead providing a smooth function relating these values to normality and disease acuity. Secondly, we have shown that these normative ranges are very specific to the subpopulations being treated going from adult to childhood and the neonatal period where the personalized interpretation of these laboratory studies is markedly different. Thirdly, we have shown that clinical setting, the grouping of tests into panels, and screening guidelines can potentially lead to overuse of laboratory tests. This automated form of EBM does not depend on an ongoing knowledge extraction process from experts; it is driven directly from aggregate physician behavior as seen in EHRs. If styles of practice change, if the meaning of particular clinical variables and their values are understood differently over time, if additional phenotypes such as genomic are introduced then the normative practice for the patient's state induced from physician behavior will automatically be changed. This study represents only a beginning in developing an automated application of physician group intelligence, similar to what has been done with “crowdsourcing” for scientific discovery in other fields [20]–[23].

There are far more sources of data that are accessible beyond laboratory data, that are driven by physician behavior and their integrated understanding of the patient's state. For example, one could examine which medications are prescribed and the number of refills included on the initial prescriptions, which procedures are ordered and the time intervals between them, how often follow-up visits are scheduled, and the number of different physicians that treat a patient. These are processes, not outcome measures, but in aggregate represent a consensus estimate.

As in other applications of group intelligence, the use of physician behavior rather than measured outcomes to drive the personalization of medical practice has some obvious risks that are built upon several underlying assumptions. The most important of these is that physicians in aggregate are well informed of the current state of the art. Further, over large populations of patients, sufficient numbers of decisions can be measured that across the varying states of patients there will be robust characterizations of the patient subpopulations. These assumptions can be tested empirically in the future by comparing physician behavior at different institutions and determining, for example, how rapidly physician behavior changes to account for the emergence of innovative and expert-approved clinical practices.

The intent of this study was not to draw conclusions about specific laboratory tests. A more detailed analysis of which tests are grouped into panels, how policies vary across different clinics, and what changes have been seen over time would be needed for that. Rather, our goal was to demonstrate that a wealth of often overlooked information about physician behavior exists in EHRs, which could provide an important source of data for future EBM research.
